# Pushing the limits of solubility prediction via quality-oriented data selection

**DOI:** 10.1016/j.isci.2020.101961

**Published:** 2020-12-17

**Authors:** Murat Cihan Sorkun, J.M. Vianney A. Koelman, Süleyman Er

**Affiliations:** 1 DIFFER - Dutch Institute for Fundamental Energy Research, De Zaale 20, 5612 AJ Eindhoven, the Netherlands; 2 CCER - Center for Computational Energy Research, De Zaale 20, 5612 AJ Eindhoven, the Netherlands; 3 Department of Applied Physics, Eindhoven University of Technology, 5600 MB Eindhoven, the Netherlands

**Keywords:** Chemistry, Analytical Reagents, Computational Chemistry, Artificial Intelligence

## Abstract

Accurate prediction of the solubility of chemical substances in solvents remains a challenge. The sparsity of high-quality solubility data is recognized as the biggest hurdle in the development of robust data-driven methods for practical use. Nonetheless, the effects of the quality and quantity of data on aqueous solubility predictions have not yet been scrutinized. In this study, the roles of the size and the quality of data sets on the performances of the solubility prediction models are unraveled, and the concepts of actual and observed performances are introduced. In an effort to curtail the gap between actual and observed performances, a quality-oriented data selection method, which evaluates the quality of data and extracts the most accurate part of it through statistical validation, is designed. Applying this method on the largest publicly available solubility database and using a consensus machine learning approach, a top-performing solubility prediction model is achieved.

## Highlights

• Consensus machine learning models perform better than singular models
• Quality-oriented data selection yields better results than using all data
• The uncertainty of test data determines the theoretical limit of a model's performance
• The concepts of actual and observed performances of solubility models are introduced

## Graphical Abstract

**Figure:**
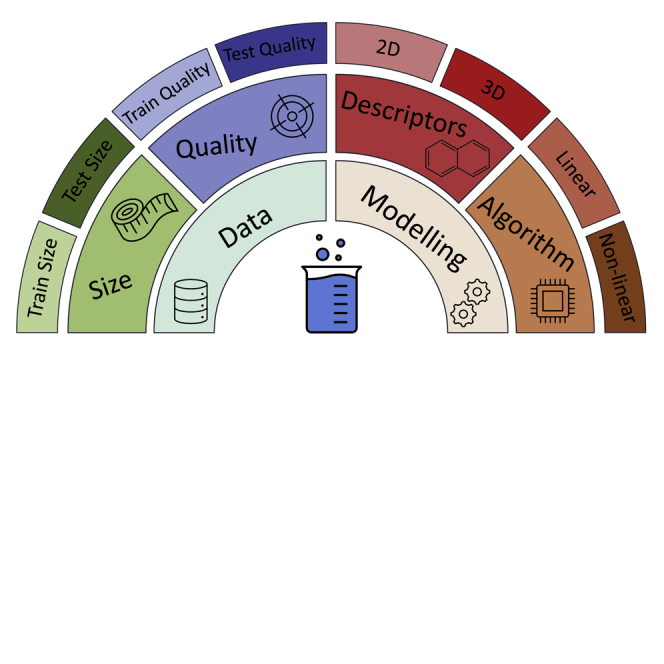

## Introduction

The solubility of chemical compounds in water is of fundamental interest, besides being a key property in the design, synthesis, performance, and functioning of new chemical motifs for various applications, including but not limited to drugs, paints, coatings, and batteries. Due to time, cost, and feasibility constraints on experimental measurements (Murdande et al., 2011), it is usually not straightforward to obtain the solubility data of compounds rapidly. Moreover, considering the vastness of chemical space, where the total number of small molecules (with up to 36 heavy atoms) is approximated to reach 10^33^ (Polishchuk et al. 2013), it is necessary to find alternative routes for the accelerated screening of candidate molecules with intended solubility values. Data-driven modeling holds the promise of making solubility predictions in a tiny fraction of a second. A data-driven model development consists of three main steps: collecting and processing train and test data, extracting and selecting key molecular descriptors, and training and testing the model.

**Figure 1:**
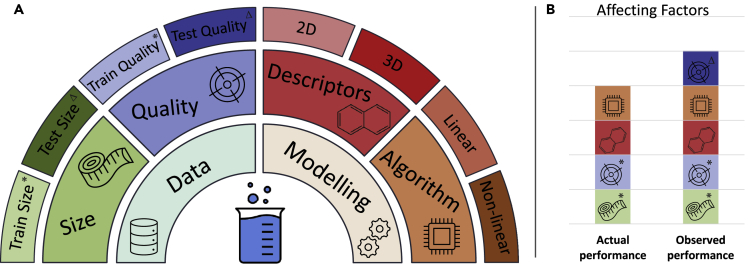
The categorization of the affecting factors for solubility predictions and their relationship with the actual and observed performances (A) The three-layered structure showing the categorization of the affecting factors on the accuracy of solubility prediction ML models. (B) The representation of affecting factors shown by the colors and symbols in Figure 1A on the actual and observed performances of solubility prediction models.

In recent years, there has been a burgeon of efforts that apply the above steps for the development of data-driven solubility prediction models. Although data-driven solubility prediction models cater for achieving results quickly, they have not yet widely been adopted in the community due to accuracy issues (Jouyban 2009). The factors that affect the performances of prediction models can be basically grouped into four categories (Haghighatlari et al., 2020): the size of data, the quality of data, the relevance of chemical descriptors, and the capability of the algorithm (Figure 1A). The first two pertain to the data and the latter two pertain to the model.

Depending on the physical domain of the problem, the above factors may vary in their significance. In the case of solubility, the paucity of measurement data, in addition to the internal errors that result from the uncertainties in experimental procedures, is well-known. Thus, the size and quality of data have priority interest when improving the performance of solubility prediction models (Tetko et al., 2001; Jorgensen and Duffy 2002; Bergstroom et al., 2004; Balakin et al. 2006; Hewitt et al., 2009; Wang and Hou 2011; Falcón-Cano et al., 2020). The latter is generally accepted as the accuracy threshold of a model. In this context, Jorgensen and Duffy stated that the accuracy of a model cannot exceed the accuracy of the experimental data (Jorgensen and Duffy 2002). Although this statement is correct, it can further be consolidated since machine learning (ML) algorithms are capable of dealing with errors in the training data (Kordos and Rusiecki 2016). To put it differently, the observed performance of a model cannot be better than the internal error of the test set. To improve the capability of solubility prediction algorithms, it is therefore important to distinguish the actual and the observed performances of a model and to comprehend the factors affecting them. Figure 1B shows a decomposition of the factors that affect the actual and observed performances of a model. We define the actual performance as the accuracy of the model that would be observed on a test set with zero internal error. In contrast, the observed performance is the accuracy of the model demonstrated on an available test set with internal error (Figure 1B). Obviously, when testing a model one can obtain only the observed performance. For instance, testing a perfect model, which by definition should predict absolute true values, on a test set with internal error of ε, will result in observed error of ε, despite the true error being zero. Therefore, the test set quality sets the theoretical limit for the observed performance of the model. In domains where high-quality data is accessible, the gap between the actual and observed performances is small enough to be ignored. However, for the case of solubility, this gap has decisive importance and should be carefully treated.

In the current work, to develop an accurate solubility prediction model, we focus on the effects of data size and data quality on the prediction performance of ML models. Starting with the design of a quality-oriented data selection method that extracts the most accurate part of the data, and applying it on AqSolDB (Sorkun et al. 2019) – the largest publicly available solubility data set that has been curated by using multiple data sources – the Aqueous Solubility Prediction Model (AqSolPred) is developed. AqSolPred shows superior test performance when compared to available models on a conventionally used benchmark data set (Huuskonen 2000). In addition to quality-oriented data selection, AqSolPred comprises a consensus of three different ML algorithms, namely Artificial Neural Network (ANN), Random Forest (RF), and Extreme Gradient Boosting (XGB). Below, we provide a detailed description of the development process, alongside the links to open-source codes and the data.

In the following paragraphs, we briefly review the principal factors that affect the accuracy of solubility predictions.

### The size of data

It is a well-known fact that increasing the number of data instances in the training set has a positive effect on the accuracy of data-driven models. For instance, Lusci et al. trained four different UG-RNN models by using datasets with 1144, 1026, 74, and 125 instances, and obtained the respective root mean squared errors (RMSEs) of 0.58, 0.60, 0.96, and 1.14 (Lusci et al. 2013). It should be noted that the size of the train and test sets yield different impacts. While the size of the training set affects the accuracy of the model, the size of the test set affects the reliable evaluation of the model's accuracy. A proper test set should be both large and diverse enough to cover the chemical space of the training set and to be minimally affected by outliers. Moreover, the solubility values of the test set should have a distribution similar to that of the training set. For example, one of the test sets (Yalkowsky and Banerjee 1992) commonly used in the literature (Tetko et al., 2001; Delaney 2004; Dearden 2006) consists of only 21 instances, which is not large enough for reliable testing. Since there had been very few solubility data publicly available, studies on solubility prediction have been limited with a few thousands of compounds for training and a few hundreds of compounds for testing (Balakin et al. 2006; Dearden 2006). With an increase in public data resources, such as AqSolDB (Sorkun et al. 2019) consisting of a diverse set of ∼10^4^ compounds, it is becoming more feasible to conduct reliable testing studies to improve the accuracies of the data-driven models.

### The quality of data

Performing high-quality solubility measurements is a difficult task due to uncertainties in experimental procedures, as explained in detail in (Avdeef, 2020). Additionally, unintentional misprints, such as the erroneous conversions of values or units while carrying them from one source to another, cause deterioration in the quality of data. Unfortunately, not all solubility data sources provide uncertainty information on individual compounds or on the complete data set. The generally accepted SD of public datasets is between 0.5 and 0.6 LogS (Jorgensen and Duffy 2002; Balakin et al. 2006). Recently, Avdeef has determined the average SD of 870 molecules from the Wiki-pS0 database as 0.17 LogS (Avdeef 2019), which is quite distant from the conceded values in literature. Therefore, we should keep in mind that the SD values are specific to data and they may differ significantly depending on the uncertainty of the measurement methods and the types of chemical compounds they contain. For example, lowly soluble compounds are extremely difficult to measure (Hewitt et al., 2009), thus the experimental errors in their measurements can be high. Accordingly, one expects that the datasets that contain many lowly soluble compounds to have high SDs. Therefore, it is essential to determine the quality of the datasets prior to the development of supervised ML models.

Similar to data size, the quality of the train and the test sets have distinct effects on the performance and therefore on the assessment of the model. Test set quality regulates the theoretical limit of observed performance (Figure 1). Therefore, to correctly evaluate the performance of a model, it is vital to use a high-quality test set. For instance, in a recent solubility prediction challenge (Llinas et al. 2020), two test sets of different qualities: high quality (SD: 0.17 LogS) and low quality (SD: 0.62 LogS), have been shared and the participants were invited to predict the solubility of compounds by using their own training data sets and methods. From a total of 37 different methods, the average RMSE for the high- and the low-quality data sets were 1.14 and 1.62 LogS, respectively. All the prediction models performed worse on the low-quality data and better on the high-quality data. This result shows the importance of test set quality on the observed performance of the models. While the test set quality affects only the observed performances of the models, the training set quality affects both the actual and observed performances. However, the internal errors of the training sets are partly compensated by capable ML algorithms depending on the size and the diversity of data. Thus, the effects of the internal errors of the training sets on the models' performances are usually smaller than the internal errors themselves.

### The relevance of chemical descriptors

Descriptors provide a mathematical representation of the chemical information contained in a compound. They are valuable inputs for data-driven models aimed at the prediction of chemical properties. Descriptors can be classified into two groups: 2D and 3D. Basically, all the descriptors that require 3D optimization of the structure are considered as 3D descriptors while the remaining are considered as 2D descriptors. There are several publicly available resources to calculate molecular descriptors (Yap 2011; Moriwaki et al., 2018). Most 2D descriptors are calculated with absolute accuracy while the 3D descriptors carry the errors of the methodological approximations they have been calculated with (Raevsky et al., 2019). Admitting that the 3D descriptors provide more detailed information, such as atomic distances and energy data of the compounds, there is yet no clear evidence about their impacts on the solubility predictions (Balakin et al. 2006; Gao et al., 2020; Yan et al., 2004; Salahinejad et al., 2013). Although a large number of chemical descriptors are available, it is usually preferred to use a modest number of relevant descriptors to avoid redundancy and overfitting issues during the training of ML models (Wang and Hou 2011).

### The capability of the algorithms

The earlier methods for solubility prediction were based on simple linear regression (LR) methods (Delaney 2004; Hansch et al. 1968; Yalkowsky and Valvani 1980; Meylan et al. 1996) and used only a few descriptors, such as lipophilicity (LogP), melting point, and molecular weight. While these methods are easy to apply and interpret, their predictive power is rather limited since the LR works only for linear dependencies. In the last years, ML algorithms, such as the variations of ANNs and tree-based ensembles, proved their ability on solving complex problems in various research fields, also including the solubility predictions (Tetko et al., 2001; Huuskonen 2000; Lusci et al. 2013; Yan and Gasteiger 2003; Schroeter et al., 2007; Tang et al., 2020). Due to their black-box nature, these algorithms are hard to interpret by humans. Moreover, they require large data sets and expert domain knowledge to circumvent overfitting issues. As ML algorithms are properly configured and fed with sufficient amount of data, they become more competent in solubility predictions. Compared to the individual models, consensus modeling that combines the predictions of different models (Todeschini et al., 2020) with an aim to compensate the weaknesses of each model, shows improved performances (Bergstroom et al., 2004; Abshear et al., 2006; Chevillard et al., 2012; Raevsky et al., 2015). Additionally, the variances in the predictions of the constituting models provide valuable information about the prediction uncertainties.

## Results

### Quality assessment of the solubility data sets

**Figure 2:**
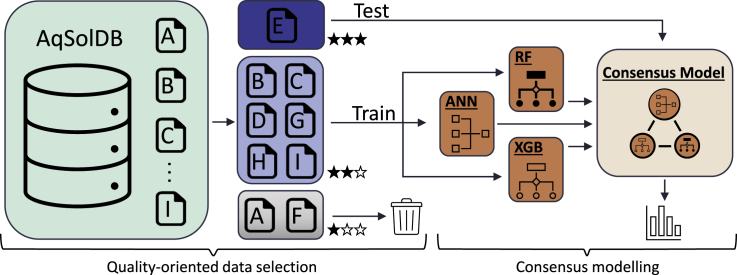
The development phases of AqSolPred The application of quality-oriented data selection method for selecting the test and training data based on their quality levels as indicated by stars (*left*). The development of the consensus model based on ANN, RF, and XGB, and its processes of training and testing (*right*).

The data selection and model development phases of the AqSolPred are shown in Figure 2. For train and test purposes, AqSolDB that merges nine different sub-datasets, named from *A* to *I*, is used (Table 1). Detailed information about the sub-data sets has been provided in (Sorkun et al. 2019), alongside the publicly accessible database (https://doi.org/10.7910/DVN/OVHAW8) and the source code including the steps for data curation (https://doi.org/10.24433/CO.1992938.v1).

**Table 1:** The SD of AqSolDB and its sub-data sets

Data set	Size	Filtered size	***N*(SD)**	**SD**
A	6110	3266	3093	0.717
B	4651	3185	1215	0.372
C	2603	1798	668	0.380
D	2115	1054	179	0.361
E	1291	1290	337	0.274
F	1210	1011	202	0.582
G	1144	363	170	0.392
H	578	148	100	0.383
I	94	62	46	0.338
All	9982	6937	–	0.495
Non-AF	6154	4399	–	0.356

**Size,** number of instances before pre-processing; **Filtered size,** number of instances after pre-processing; ***N*(SD),** total number of multiple values used to calculate SD; **SD,** standard deviation.

As explained above, the train and test data affect the actual and observed performance of the models differently. Therefore, instead of using all available data directly, we applied a quality-oriented selection procedure for the training and test data. We determined the quality of each sub-dataset in terms of the SD of multi-lab measurements as described in the Methods. The total number of multi-lab measurements (*N* (SD)) and the calculated SDs are shown in Table 1. The SDs of the nine sub-data sets vary significantly, with numerical values between 0.274 and 0.717 LogS. The data set E has the lowest SD and therefore is considered to contain the highest quality data. Adversely, the data sets *A* and *F* have the largest SDs. The SDs of the remaining data sets are close to each other and all are <0.4 LogS.

### Selection of the test and the training data sets

**Figure 3:**
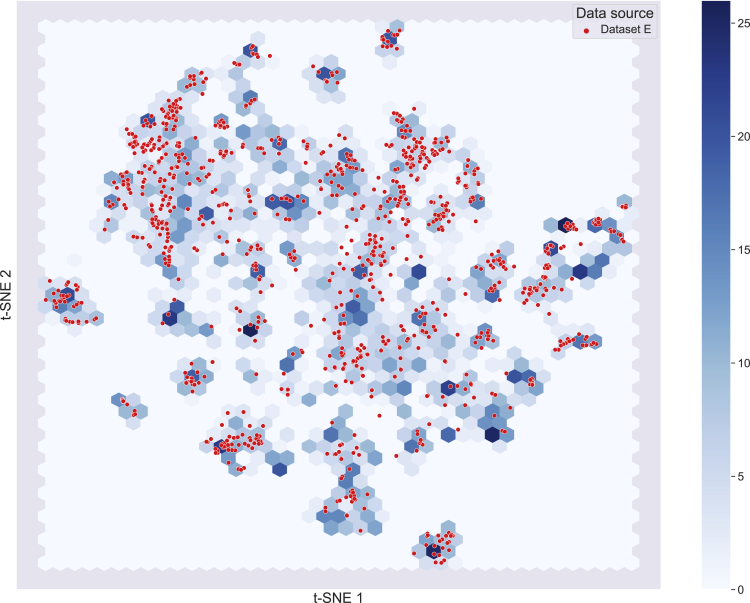
Visualization of the chemical space covered by the training and test data The chemical space is visualized by the t-SNE dimensionality reduction technique. Blue hexagons show the chemical space that is covered by the training data, whereas the red dots show the test instances in the chemical space. The color scale on the right shows the density of molecules found in the hexagons.

For a proper evaluation of the model, the observed performance of the model should approach the actual performance as explained above. Therefore, the test data should be of the highest possible quality. Additionally, it should be large enough to cover the chemical space of the training set. We selected dataset *E* as the test set since it has the highest quality among the sub-data sets. It is important to note that, data set *E* is also known as the Huuskonen data set, which is commonly used in literature as a benchmark data set. Using the t-distributed stochastic neighbor embedding (t-SNE) dimensionality reduction technique (Maaten and Hinton 2008), we validated that dataset *E* largely covers a reduced chemical space of the training data (Figure 3). We also validated that the distribution of the solubility values of data set *E* is compatible with the training set (Figure S1). After reserving data set *E* as the test set, we also removed the two sub-data sets, *A* and *F*, with large SDs. Using the remaining data sets and the curation algorithm described in (Sorkun et al. 2019), a high-quality training set, *non-AF*, is obtained. The SD of the *non-AF* dataset has been calculated by incorporating the SDs of the constituent sub-datasets. For comparison, we also calculated the SD of the entire AqSolDB, namely the *All*, using the same procedure (Table 1).

### Effect of quality and size of the training set

**Figure 4:**
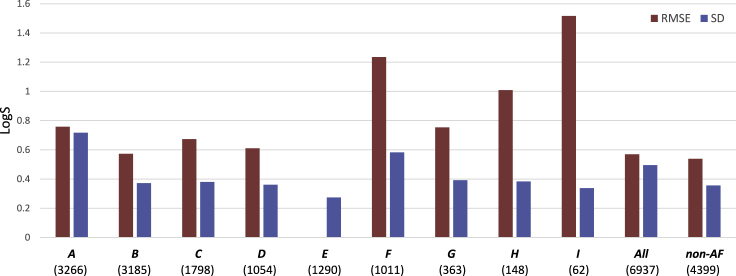
The quality and accuracy comparison of the sub-data sets Blue bars show the SD of sub-data sets, whereas the red bars show the test performances (RMSE) on data set *E* of the models that have been trained by that sub-data set. Both the SD and RMSE are given units of LogS. The total number of data instances that have been used to train the models are shown for each sub-data set.

As discussed above, both the size and the quality of training set are positively correlated with a model's accuracy. However, quality-oriented data selection decreases the size of the data while increasing the quality. To analyze the trade-off between size and quality, we developed separate models for each solubility sub-data set. For a fair comparison, we trained sub-data sets with the same combinations of feature selection methods and ML algorithms explained in the Methods. We selected the best configurations based on 10-fold cross-validation performances of each of the sub-data sets. We trained the final models using their best configurations and the entire training data. After ensuring that no test compounds were used in the training process (see Methods), we tested the performances of the final models against the test data set *E* (Figure 4). To understand the effect of data quality in predictions, we compared the datasets of similar size, *A-B* and *D-F*, and found that those having higher quality perform significantly better than those having lower quality. To understand the effect of size, we compared the data sets of similar quality. First, we compared data sets *B*, *C*, and *D*, with 3185, 1798, and 1054 instances, respectively. The test performances of these three datasets are very close, within ∼0.1 LogS (Figure 4). Secondly, we compared datasets *G*, *H*, and *I*, whose qualities are similar but the sizes are 363, 148, and 62, respectively. This time the size effect is more obvious, as the accuracy decreases when the size of the data becomes smaller (Figure 4). Despite having the lowest SD within the group of training sub-data sets, *I* shows the lowest accuracy due to its small size. According to these results, we conclude that the data size is more influential on small-sized data sets with a few hundred or fewer instances, while the data quality is more effective on large-sized datasets with thousands of instances.

The quality-oriented data selection data set, *non-AF*, shows superior performance among all data sets by virtue of its quality, despite the fact that this data set has 2617 fewer instances than the largest data set *All*. So far all the models have been developed without using any compounds from data set *E*. To quantify the impact of including this high-quality data, in a new experiment we included data set *E* into the training process. We applied the leave-one-out (LOO) cross-validation method and left out a single compound at a time from dataset *E* for validation and included the remaining compounds in the training data. This process was repeated for each molecule in data set *E*. As expected, the inclusion of data set *E* improved the performance as shown by the bottom two rows in Table 2. Furthermore, we conducted experiments by oversampling the highest quality data, but since this did not result in noteworthy improvements we have not included them here.

**Table 2:** Comparison of AqSolPred to literature results

Year	Model	Method	Total size	Test size/method	MAE	RMSE	R2	Reference
2000	Huuskonen	ANN	1294	413	–	0.600	0.92	Huuskonen (2000)
2000	Huuskonen	MLR	1294	413	–	0.710	0.88	Huuskonen (2000)
2001	Tetko	ANN	1291	412	–	0.620	0.91	Tetko et al., (2001)
2003	Yan	MLR	1294	496	0.680	0.790	0.82	Yan and Gasteiger (2003)
2003	Yan	ANN	1294	496	0.490	0.590	0.92	Yan and Gasteiger (2003)
2004	Delaney^a^	MLR	1290	1290	0.685	0.876	0.71	Delaney (2004)
2004	Hou	MLR	1294	412	0.520	0.630	0.90	Hou et al., (2004)
2007	Schroeter	GP	1290	3 fold CV	0.412	0.579	–	Schroeter et al., (2007)
2007	Schroeter	RR	1290	3 fold CV	0.586	0.996	–	Schroeter et al., (2007)
2007	Schroeter	SVM	1290	3 fold CV	0.431	0.600	–	Schroeter et al., (2007)
2007	Schroeter	RF	1290	3 fold CV	0.485	0.660	–	Schroeter et al., (2007)
2012	Ali^a^	MLR	1290	1290	0.728	0.940	0.73	Ali et al., (2012)
2013	Lusci	UG-RNN	1026	10-fold CV	0.460	0.600	0.91	Lusci et al. (2013)
2016	Filter-it^a^	MLR	1290	1290	0.893	1.154	0.68	Daina et al. (2017)
2018	Bjerrum	ANN	1297	10-fold CV	–	0.650	0.90	Bjerrum and Sattarov, 2018
2020	Tang	MPN	1310	10-fold CV	–	0.661	–	Tang et al., 2020
2020	AqSolPred	Consensus	1290	1290	**0.397**	**0.539**	**0.93**	–
2020	AqSolPred	Consensus	1290	LOO	**0.348**	**0.483**	**0.94**	–

**ANN,** artificial neural networks; **MLR,** multiple linear regression; **GP,** Gaussian processes; **RR,** Ridge regression; **SVM,** support vector machine; **RF,** Random forest; **UG-RNN,** undirected graph-recursive neural networks; **MPN,** message parsing neural network; **consensus,** an ensemble of ANN, RF, and XGB.

a Results collected from SwissADME web tool (Daina et al. 2017).

These results show that both the quality and the size of data have major impacts on the solubility prediction performances of the ML models. Moreover, instead of direct use of all the available data for training, a quality-oriented data selection method empowers the model.

### Effect of descriptors and algorithms

We used a total of 123 2D descriptors for which the groupings, sizes, and use cases from literature are shown in Table 3.

**Table 3:** The groupings of chemical descriptors

Group	Size	References
Atom-based	19	Lusci et al. (2013); Avdeef, 2020; [Bibr bib43][Bibr bib34]; Tang et al., 2020; Hou et al., (2004)
Ring-based	6	Avdeef, 2020; Yan et al., 2004; Tang et al., 2020
Bond-based	9	Jorgensen and Duffy (2002); Lusci et al. (2013); Delaney (2004); [Bibr bib4][Bibr bib43]; Tang et al., 2020; Raevsky et al., 2015
LogP	1	Lusci et al. (2013); Delaney (2004); Avdeef, 2020; [Bibr bib43][Bibr bib34]; Raevsky et al., 2015; Ali et al., (2012)
Topological	18	Jorgensen and Duffy (2002); Huuskonen (2000); Avdeef, 2020; [Bibr bib43][Bibr bib34]; Raevsky et al., 2015; Ali et al., (2012)
E-state indices	70	Avdeef, 2020, Huuskonen, 2000, Tetko et al., 2001

To pick out a minimum number of relevant descriptors, we independently applied the LASSO and PCC feature selection methods as described in the Methods. The definitions and the correlation matrix of these descriptors are shown in Table S1 and Figure S2, respectively. The cross-validation results of the various configurations show that the LASSO performs slightly better than the PCC. Using the former method, a total of 58 descriptors have been selected.

Trained on each of the data sets, a consensus model that combines three different ML algorithms (ANN, RF, and XGB) as described in Methods, exceeds the performance of any of the singular models that have been trained by a single algorithm. Also importantly, using a consensus model it is possible to collect additional uncertainty information, whereas using the individual algorithms independently does not provide this information. This is because the SDs from different model predictions are good indicators for the uncertainties observed in the final predictions. The configurations of the different ML models and their results are shown in Tables S2, S3, S4, S5, S6, S7, S8, S9, S10 and S11.

### Performance comparisons of the model with the literature

The AqSolPred shows the highest accuracy on the Huuskonen data set (i.e. data set *E*), when compared to the available results from the literature on solubility predictions (Table 2). Due to the differences in pre-processing steps, the total number of data instances that have been used by each method differs slightly as shown in Table 2. Furthermore, some studies have used cross-validation techniques while others have divided data into train and test sets.

## Discussion

A cardinal result of the current study is the differentiation of actual and observed performances of the solubility models. Because the observed performance is highly sensitive to the quality of the test set, when the test data contains high uncertainty, the difference between actual and observed performances becomes more pronounced. Therefore, it is imperative to use high-quality data in testing to obtain an observed performance close to the actual performance of a model. For this reason, the quality assessment prior to training and testing experiments constitutes a vital step. The generally employed assumptions on the SDs of experimental datasets (e.g. such as up to 0.6 LogS error) are fuzzy and they do not necessarily reflect the true quality of data sets (see Table 1). Instead, comparing multi-lab measurement data of compounds provides a way to estimate the solubility data quality. For instance, in the current study, we collected a total of 6010 multi-lab measurements on 2236 unique compounds from nine different sources. We matched the compounds based on their InChIKeys, a safe way to identify the same compounds. Considering that the different datasets may contain compounds from the same source, as an early procedure, the duplicates should be identified to ensure the usage of the same information only once in the quality estimation step. As an example, we classified the compounds as duplicates if they have the same InChIKey and their measured solubilities are within 0.01 LogS, as described in (Sorkun et al. 2019). An added value of comparisons between multi-lab values, next to that of determining the quality of the data sets, is the detection of outliers in data, such as the ones caused by misprints.

A second conclusion is the impact of training size on the accuracy of data-driven models. We found that, regardless of their quality, the small-sized data sets do not include the generic information to address the solubility problem and they do not adequately cover the chemical space of the test data. Therefore, we recommend that extra care should be taken when reaching conclusions based on models that have been trained with small-sized data sets.

Data diversity is another important concept that designates the applicability domain of ML models. In addition to being sufficiently large as explained above, a good training set should also have a high ratio of the data size over the chemical diversity of compounds. In the case of the test data, it should cover the chemical domain defined by the training set. Visualizing the data in two-dimensions allows for inspecting to what extend the test set covers the chemical compound space of the training set. Dimensionality reduction methods (e.g. t-SNE (Maaten and Hinton 2008) and UMAP (McInnes et al. 2018)) provide interpretable 2D graphs by clustering the chemical compounds based on their local similarities. Defining the chemical space based on tailored similarities and using only the relevant descriptors of target properties, provides a better representation than using arbitrary similarities such as the predefined fingerprints (Gute et al., 2002).

During the prediction of aqueous solubility data of compounds here, the observed superior performance of a consensus model over the singular models promises that there is still room for algorithmic improvements to further improve the accuracies in solubility predictions of the compounds. When building a consensus model, increasing the number of constituent algorithms would generate more accurate predictions by facilitating the elimination of the outliers before merging the prediction results. Moreover, the uncertainty information obtained from multiple predictions would be more reliable. Lastly, since they are modeling the problem from different aspects, bringing fundamentally diverse algorithms into play would provide better results compared to using the same stochastic algorithm multiple times with different initializations.

In summary, applying a quality-oriented data selection method, employing 58 LASSO-selected 2D descriptors and an ensemble of advanced ML algorithms, we developed the AqSolPred, a high-caliber solubility prediction model.

### Limitations of the study

The SDs of the data sets are calculated using the available multi-lab values. Since the accuracy of the SDs will depend on the number of multi-lab measurement data, the calculated SDs may differ from the real SDs. The risk is higher when only a few multi-lab measurements are present.

The performance of AqSolPred is compared with models from literature as based on the published reports or generated results by using the online tools. Despite the fact that all studies considered in the present study were tested on the same dataset, due to the black-box nature of tools or the missing descriptions for training and testing processes, it is not always straightforward to make exact comparisons between the methodological aspects of the different models.

### Resource availability

#### Lead contact

Further information and requests about AqSolPred should be directed to and will be fulfilled by the lead contact, Süleyman Er (s.er@differ.nl).

#### Materials availability

This study did not produce any new molecules or materials.

#### Data and code availability

The reproducibility of the AqSolPred can be verified by executing the provided scripts on Code Ocean (https://doi.org/10.24433/CO.3467849.v2). The freely accessible AqSolPred web tool is reachable at: https://www.amdlab.nl/aqsolpred/.

## Methods

All methods can be found in the accompanying Transparent Methods supplemental file.

## Supplemental information

Document S1. Transparent methods, Figures S1and S2, and Table S1

Table S2. The results of models trained with dataset non-AF and tested on dataset E, related to Figure 4

Table S3. The results of models trained with dataset All and tested on dataset E, related to Figure 4

Table S4. The results of models trained with dataset A and tested on dataset E, related to Figure 4

Table S5. The results of models trained with dataset B and tested on dataset E, related to Figure 4

Table S6. The results of models trained with dataset C and tested on dataset E, related to Figure 4

Table S7. The results of models trained with dataset D and tested on dataset E, related to Figure 4

Table S8. The results of models trained with dataset F and tested on dataset E, related to Figure 4

Table S9. The results of models trained with dataset G and tested on dataset E, related to Figure 4

Table S10. The results of models trained with dataset H and tested on dataset E, related to Figure 4

Table S11 The results of models trained with dataset I and tested on dataset E, related to Figure 4
