# Supplementary material for: Pushing the limits of solubility prediction via quality-oriented data selection
Source: iScience. 2020 Dec 17;24(1):101961. doi: 10.1016/j.isci.2020.101961 (PMC7788089; doi:10.1016/j.isci.2020.101961)
Supplement: Document S1. Transparent methods, Figures S1and S2, and Table S1 [file mmc1.pdf]

iScience, Volume *24*

Supplemental Information

Pushing the limits of solubility prediction via quality-oriented data selection

Murat Cihan Sorkun, J.M. Vianney A. Koelman, and Süleyman Er

Transparent Methods

Quality-oriented data selection

Quality-oriented data selection identifies the quality of datasets by calculating the deviations in the multi-lab experimental measurements of the compounds. Using the quality information, the highest quality dataset is reserved as the test set and the poor quality datasets are removed from the training set. To assess the quality of each dataset, the following steps have been applied:

- Compounds that have multi-lab measurement data have been identified.
- The average of the measured solubility values of compounds have been calculated.
- The deviations of measurement data from the average values have been calculated.
- The SDs of the constituting datasets have been calculated.

The SDs for each dataset (from A to I) have been calculated using Eq. 1:

$$SD = \sqrt{\frac{1}{n}\sum_{i=1}^{n} x_i - \bar{x}} \tag{1}$$

where n is the total number of compounds that have multi-lab measurement data, x_i is the experimentally measured solubility value of compound i, and $\bar{x}$ is the average of multi-lab solubility values of the compound.

The SDs of the combinatorial datasets (i.e. "*non-AF*" and "*All*") have been calculated using Eq. 2:

$$SD = \frac{1}{N}\sum_{j=1}^{Z} SD_j T_j \tag{2}$$

where N is the total number of compounds in the dataset, Z is the total number of constituent datasets, SD_j is the SD of dataset j, and T_j is the total number of compounds that have been included from dataset j.

Data pre-processing

To prepare the datasets for training, we removed the compounds from datasets when they met any of the following criteria:

- The compound exists in the test set (dataset *E*).
- The compound does not contain carbon atom.
- The compound contains adjoined mixtures.
- The compound contains charged atoms.

The remaining numbers of compounds found in each training sub-dataset, obtained after the completion of data pre-processing, have been shown in Table 1 (Filtered Size).

Descriptor selection

To generate the molecular descriptors, we used the Mordred Python package [1]. Currently, there are more than 1800 2D and 3D descriptors in the Mordred catalog. To determine the most relevant descriptors, we applied the following feature selection methods:

- **Least absolute shrinkage and selection operator (LASSO):** A regression analysis method that enhances the prediction accuracy and interpretability of the statistical model. To learn the best descriptors (i.e. variables) the LASSO regularization eliminates the irrelevant descriptors by forcing their coefficients to zero.
- **Pearson correlation coefficient (PCC):** Selects the descriptors that have PCC with LogS higher than a defined threshold parameter.

For both methods, we tested different parameter sets that change the strictness of selections. The results of these different configurations are provided in Table S2-S11.

Out of the generated 123 descriptors using Mordred, 58 have been selected by LASSO regularization. The correlation matrix of the selected chemical descriptors is shown in Figure S2. The complete list of the selected descriptors, including their names and descriptions, are shown in Table S1.

Machine learning algorithms

We employed the following ML algorithms in combination with the scikit-learn and xgboost Python packages.

- Artificial neural network (ANN)
- Random forest (RF)
- Extreme gradient boosting (XGB)

ANN is a network consisting of several layers that are connected to each other through the neurons it contains. ANN learns non-linear functions by modifying the coefficients between neurons via a back-propagation algorithm. In the current work, the ANN configuration employs single hidden layer with 500 neurons and a *tanh* activation function. RF is an ensemble of decision trees that use bootstrap aggregating of the instances and a random sampling of the features. Our RF configuration consists of 1000 trees with the maximum depth. XGB is a regularized gradient boosting algorithm that creates a strong learner from an ensemble of many weak trees that are trained sequentially. Our XGB configuration consists of 1000 trees with a maximum depth of six. Other parameters of the models are used with their default values. Lastly, our consensus model is based on a combination of the above three ML models and an arithmetic averaging of the predictions by these models.

Configuration of the AqSolPred

The best performing AqSolPred model has been achieved by using the following configuration:

- **Training set:** *non-AF* (4399 data instances)
- **Features:** 58 2D descriptors as selected by LASSO with $\alpha = 0.01$
- **ML Algorithm:** A consensus of ANN, RF, and XGB models

Chemical space visualization

We used tailored similarity for the visualization of the chemical space based on 58 LASSO-selected descriptors. We applied t-SNE from scikit-learn Python package to reduce the data into two-dimensions with the following two parameters, while the remaining parameters are used with their default values:

- **Perplexity:** 50
- **Random state:** 1

Supplemental Figures

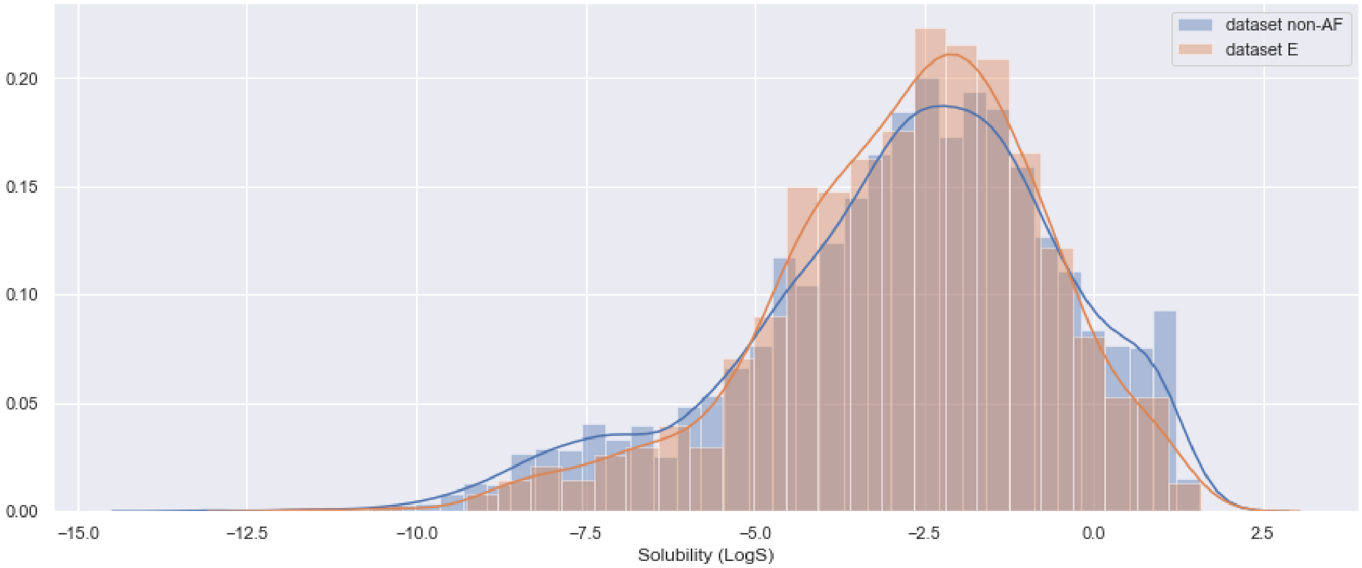

Figure S1. The normalized distribution of solubility for the train dataset (*non-AF*) and the test dataset (*E*), Related to Figure 3.

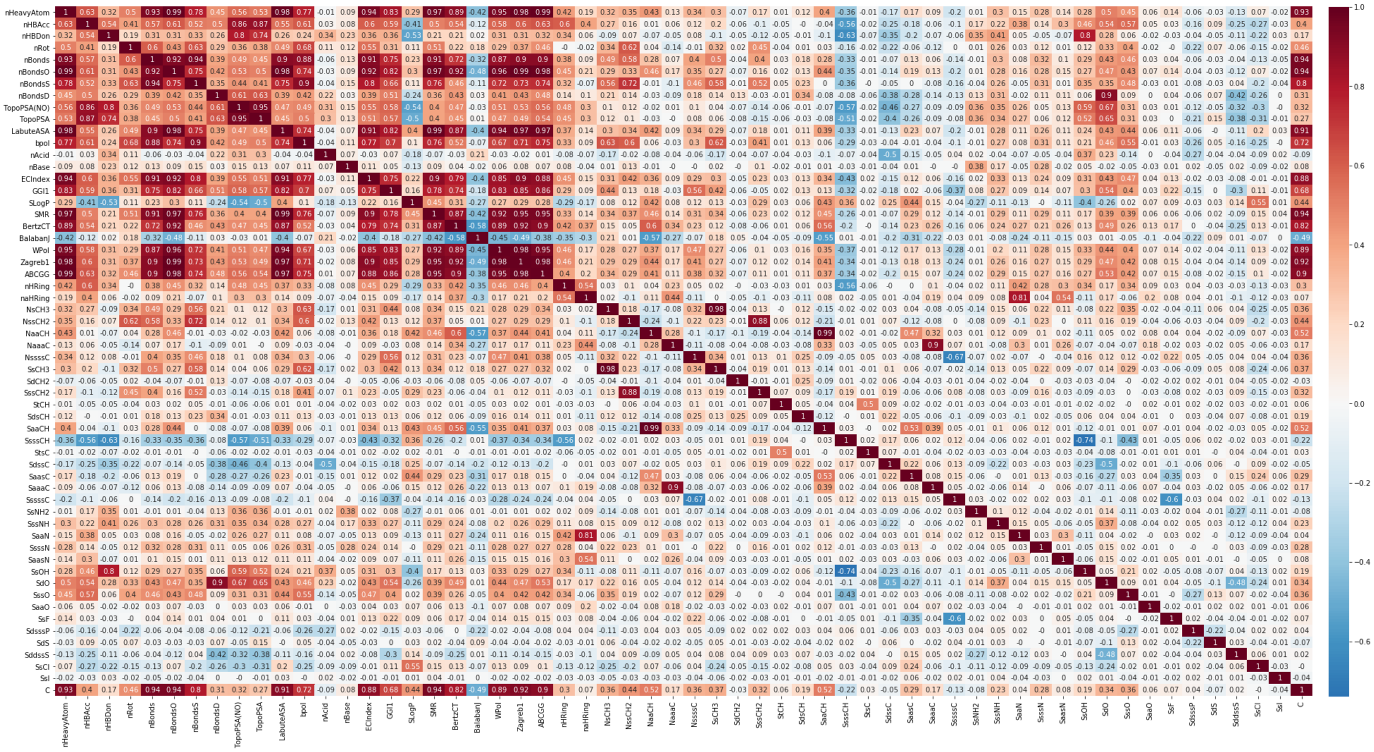

Figure S2. The correlation matrix of a total of 58 LASSO-selected chemical descriptors, Related to Table 3.

Supplemental Table

Table S1. The names and descriptions of a total of 58 LASSO-selected descriptors, Related to Table 3.

ID	Name	Description	ID	Name	Description
1	nHeavyAtom	number of heavy atoms	30	NssssC	number of ssssC
2	nHBAcc	number of hydrogen bond acceptor	31	SsCH3	sum of sCH3
3	nHBDon	number of hydrogen bond donor	32	SdCH2	sum of dCH2
4	nRot	rotatable bonds count	33	SssCH2	sum of ssCH2
5	nBonds	number of all bonds in non-kekulized structure	34	StCH	sum of tCH
6	nBondsO	num of bonds connecting to heavy atom in non-kekulized structure	35	SdsCH	sum of dsCH
7	nBondsS	number of single bonds in non-kekulized structure	36	SaaCH	sum of aaCH
8	nBondsD	number of double bonds in non-kekulized structure	37	SsssCH	sum of sssCH
9	TopoPSA(NO)	topological polar surface area (use only nitrogen and oxygen)	38	StsC	sum of tsC
10	TopoPSA	topological polar surface area	39	SdssC	sum of dssC
11	LabuteASA	Labute's Approximate Surface Area	40	SaasC	sum of aasC
12	bpol	bond polarizability	41	SaaaC	sum of aaaC
13	nAcid	acidic group count	42	SssssC	sum of ssssC
14	nBase	basic group count	43	SsNH2	sum of sNH2
15	ECIndex	eccentric connectivity index	44	SssNH	sum of dNH
16	GGI1	1-ordered raw topological charge	45	SaaN	sum of aaN
17	SLogP	Wildman-Crippen LogP	46	SsssN	sum of sssN
18	SMR	Wildman-Crippen MR	47	SaasN	sum of aasN
19	BertzCT	Bertz CT	48	SsOH	sum of sOH
20	BalabanJ	Balaban's J index	49	SdO	sum of dO
21	WPol	Wiener polarity index	50	SssO	sum of ssO
22	Zagreb1	Zagreb index (version 1)	51	SaaO	sum of aaO
23	ABCGG	atom-bond connectivity index	52	SsF	sum of sF
24	nHRing	hetero ring count	53	SdsssP	sum of dsssP
25	naHRing	aromatic hetero ring count	54	SdS	sum of dS
26	NsCH3	number of sCH3	55	SddssS	sum of ddssS
27	NssCH2	number of ssCH2	56	SsCl	sum of sCl
28	NaaCH	number of aaCH	57	SsI	sum of sI
29	NaaaC	number of aaaC	58	C	C atoms count

References

[1] Moriwaki, H., Tian, Y. S., Kawashita, N. & Takagi, T. Mordred: a molecular descriptor calculator. *Journal of cheminformatics* **10**, 4 (2018).
